# Supplementary material for: Exploring the mitochondrial genomes and phylogenetic relationships of trans-Andean Bryconidae species (Actinopterygii: Ostariophysi: Characiformes)
Source: PLoS One. 2024 Aug 27;19(8):e0300830. doi: 10.1371/journal.pone.0300830 (PMC11349099; doi:10.1371/journal.pone.0300830)
Supplement: Attachment — Submitted filename: Response to Reviewers.docx [file pone.0300830.s001.docx]

Dear Editor

We greatly appreciate the detailed and constructive feedback from the reviewers. We have revised the manuscript according to their valuable recommendations, resulting in significant improvements to our paper.

We hope that the manuscript is now suitable for publication in Plos One.

PONE-D-24-08613

Exploring the mitochondrial genomes and phylogenetic relationships of eleven Bryconidae species

PLOS ONE

Dear Dr. Márquez,

Thank you for submitting your manuscript to PLOS ONE. After careful consideration, we feel that it has merit but does not fully meet PLOS ONE’s publication criteria as it currently stands. Therefore, we invite you to submit a revised version of the manuscript that addresses the points raised during the review process. Please see my own comments an the reviews of two reviewers below.

Please submit your revised manuscript by May 30 2024 11:59PM. If you will need more time than this to complete your revisions, please reply to this message or contact the journal office at [plosone@plos.org](mailto:plosone@plos.org). When you're ready to submit your revision, log on to <https://www.editorialmanager.com/pone/> and select the 'Submissions Needing Revision' folder to locate your manuscript file.

Please include the following items when submitting your revised manuscript:

- A rebuttal letter that responds to each point raised by the academic editor and reviewer(s). You should upload this letter as a separate file labeled 'Response to Reviewers'.
- A marked-up copy of your manuscript that highlights changes made to the original version. You should upload this as a separate file labeled 'Revised Manuscript with Track Changes'.
- An unmarked version of your revised paper without tracked changes. You should upload this as a separate file labeled 'Manuscript'.

If you would like to make changes to your financial disclosure, please include your updated statement in your cover letter. Guidelines for resubmitting your figure files are available below the reviewer comments at the end of this letter.

If applicable, we recommend that you deposit your laboratory protocols in [protocols.io](http://protocols.io/) to enhance the reproducibility of your results. Protocols.io assigns your protocol its own identifier (DOI) so that it can be cited independently in the future. For instructions see: <https://journals.plos.org/plosone/s/submission-guidelines#loc-laboratory-protocols>. Additionally, PLOS ONE offers an option for publishing peer-reviewed Lab Protocol articles, which describe protocols hosted on [protocols.io](http://protocols.io/). Read more information on sharing protocols at <https://plos.org/protocols?utm_medium=editorial-email&utm_source=authorletters&utm_campaign=protocols>.

We look forward to receiving your revised manuscript.

Kind regards,

Roberto E. Reis, Ph.D.
Academic Editor
PLOS ONE

Journal Requirements:

When submitting your revision, we need you to address these additional requirements.

1. Please ensure that your manuscript meets PLOS ONE's style requirements, including those for file naming. The PLOS ONE style templates can be found at 
<https://journals.plos.org/plosone/s/file?id=wjVg/PLOSOne_formatting_sample_main_body.pdf> and 
<https://journals.plos.org/plosone/s/file?id=ba62/PLOSOne_formatting_sample_title_authors_affiliations.pdf>

2. Please include your full ethics statement in the ‘Methods’ section of your manuscript file. In your statement, please include the full name of the IRB or ethics committee who approved or waived your study, as well as whether or not you obtained informed written or verbal consent. If consent was waived for your study, please include this information in your statement as well.

Additional Editor Comments:

The manuscript is mostly well written, but its title and abstract fits better in a paper on genetic data than in a phylogenetic contribution. The authors have somewhat mixed concepts and failed to explore and extract the full potential of the data so that this manuscript could be considered a phylogenetic contribution. Both reviewers mentioned problems related to be above and presented excellent sugestions, which I agree with.

We have edited these sections as described below.

Further on these general issues, I have a few format corrections:
1. Only cite species authorities (author, year) the first time a name appears in the manuscript.

Done.

2. Chose and standradize the language the geographic descriptors. You use both Spanish (quebrada, río) and english (River).

As suggested by Reviewer #1, we have removed this information.

[Note: HTML markup is below. Please do not edit.]

Reviewers' comments:

Reviewer's Responses to Questions

**Comments to the Author**

1. Is the manuscript technically sound, and do the data support the conclusions?

The manuscript must describe a technically sound piece of scientific research with data that supports the conclusions. Experiments must have been conducted rigorously, with appropriate controls, replication, and sample sizes. The conclusions must be drawn appropriately based on the data presented.

Reviewer #1: Yes

Reviewer #2: Yes

2. Has the statistical analysis been performed appropriately and rigorously?

Reviewer #1: I Don't Know

Reviewer #2: Yes

3. Have the authors made all data underlying the findings in their manuscript fully available?

The [PLOS Data policy](http://www.plosone.org/static/policies.action#sharing) requires authors to make all data underlying the findings described in their manuscript fully available without restriction, with rare exception (please refer to the Data Availability Statement in the manuscript PDF file). The data should be provided as part of the manuscript or its supporting information, or deposited to a public repository. For example, in addition to summary statistics, the data points behind means, medians and variance measures should be available. If there are restrictions on publicly sharing data—e.g. participant privacy or use of data from a third party—those must be specified.

Reviewer #1: Yes

Reviewer #2: Yes

4. Is the manuscript presented in an intelligible fashion and written in standard English?

PLOS ONE does not copyedit accepted manuscripts, so the language in submitted articles must be clear, correct, and unambiguous. Any typographical or grammatical errors should be corrected at revision, so please note any specific errors here.

Reviewer #1: Yes

Reviewer #2: Yes

5. Review Comments to the Author

Please use the space provided to explain your answers to the questions above. You may also include additional comments for the author, including concerns about dual publication, research ethics, or publication ethics. (Please upload your review as an attachment if it exceeds 20,000 characters)

Reviewer #1: Comments on MS PONE-D-24-08613 “Exploring the mitochondrial genomes and phylogenetic relationships of eleven Bryconidae species” by Edna Judith Marquez, Daniel Alfredo Gómez-Chavarria & Juan Fernando Alzate.

That’s an interesting contribution that improves our understanding of the phylogenetic relationships within the family Bryconidae, especially because it focuses transandean taxa, that have been neglected in previous phylogenies of the family. I consider that the manuscript has a lot of merit and should be published, but I have a few suggestions for its improvement, that follow below.

Title: I suggest changing “eleven” for “Transandean” to emphasize what constitutes to be the main contribution of the present MS regarding the knowledge of Bryconidae.

Done. Now, considering comments of both reviewers: “Exploring the mitochondrial genomes and phylogenetic relationships of trans-Andean Bryconidae species (Actinopterygii: Ostariophysi: Characiformes)”

Abstract, l. 31-33: It would be preferrable to treat the genus Salminus as another clade as this genus has been only considered as closely related to Brycon after phylogenies using molecular data have demonstrated it. The fact that Salminus is a cis/transandean lineage is known since the description of Salminus affinis by Steindachner in 1877, as there was never any doubt that Salminus is monophyletic. It would be better simply to state that Brycon rubricauda is sister to a clade including Salminus and Brycon species mostly from cis-andean systems but including a species from the Magdalena/Cauca, B. moorei. Notice also that Brycon henni and a Brycon of the B. atrocaudatus species complex occur at the Magdalena/Cauca basin, so the generalization that Bryconidae from the Magdalena/Cauca group with cisandean taxa is incorrect.

Done. Now: “The other clade grouped the trans-Andean species from the Magdalena-Cauca Basin *Brycon moorei* and *Salminus affinis*, with their respective cis-Andean congeners (found in eastern South America), with Brycon rubricauda as its sister clade”

In Discussion: “On the other hand, in the cis-Andean + Magdalena-Cauca Basin (*Brycon moorei* and *Salminus affinis*) clade, the remaining eight species were clustered, displaying 100% UFB support, with 100% and 49.5% concordance factors for genes and sites, respectively.”

Introduction, P. 3, l. 55-56. There are actually 44 Brycon species currently considered as valid. The authors should cite the much more updated and broad revision of the cisandean Brycon by Lima (2017) instead of Lima (2004) for that information, since the latter reference actually mentions 41 valid species of Brycon, which was the number of species recognized at that time, and it is only a species description, not a revision.

Done

P.3, L. 56-57: Lima (2004) does not state at any point that Brycon shows its greater diversity at transandean rivers. The authors should instead cite Lima (2017), which mentions at p. 4 that half of the Brycon species currently recognized as valid are transandean, so the diversity of the genus is equally divided between the transandean and cisandean South America. It is probably true that the area between Panama and coastal northern Peru concentrates the largest diversity in the family per area, the problem is that this need to be clearly demonstrated, but it hasn’t so far and at the moment there are no references that the authors could cite to support that assertion.

Done. We removed “showing its greater taxonomic diversity in Panama and the Colombian and Ecuadorian trans-Andean rivers [15]”

Material and methods, L. 83-93: Not sure that the authors need to go into this detail in describing their samples. A table as an additional file would suffice for that.

P. 14, L. 305-314: The authors does not address the fact that their results are affected by the obvious fact that they are just sampling a small fraction of the diversity of the family, and much less terminals than the largest published phylogenetic study of the family (Abe et al., 2014). They should be more cautious when inferring relationships and remind the readers that difference in results may be simply a result of sampling bias. For example, there is very compelling morphological evidence that Brycon henni forms a monophyletic clade with B. stolzmanni and B. coxeyi (see Lima, 2017), two species that have not being analysed in the present study (nor in Abe et al., 2014). There is no need for recommending a “taxonomic revision of the group” as it is well-know that the taxonomy of transandean Brycon is in a state of disarray, so the authors are making an unnecessary recommendation.

Now: “Since only 19 mitogenomes corresponding to 13 species of Bryconidae were included in our analysis, the phylogenetic relationships found may be influenced by an incomplete sampling. Despite this limitation, the current analysis recovers the phylogenetic relationships previously reported and offers new insights into the origin and diversification of Bryconidae groups. The trans-Andean species from the Pacific slope drainages were grouped, with *B. meeki* and *B. chagrensis* as sister species (UFB: 100, GCF: 100; SCF: 85.3), while *B. oligolepis* was clustered with *Chilobrycon* (UFB: 100, GCF: 100, SCF 57.6). *Brycon henni* appears as an ancestral lineage in this last clade, also with 100% UFB support. Previously described phylogenetic relationships of *B. chagrensis* as the sister clade of *Chilobrycon* and *B. henni* [14] indicate that *B. chagrensis* + *B. meeki* is also a sister clade of (*B. henni* + (*Chilobrycon* + *B. oligolepis*)).

P. 15, l. 326-329: Salminus affinis is only recovered as sister of S. brasiliensis because the authors have not sampled any other Salminus species! See Abe et al. (2104) and Machado et al. (2018) (which should be cited by the authors), and check the discussion on the phylogeny of Salminus by Lima (2022). As noticed above, the authors are mistakenly assuming relationships without considering that their study, despite its merits in including taxa absent from other phylogenetic analyses of the family, it nonetheless only includes a small proportion of the diversity of the family and consequently, much caution should be used in inferring relationships.

We removed “and *S. affinis* is a sister species of *S. brasiliensis*” and include an explanation to clarify this idea.

Now: “Furthermore, *B. moorei* was found to be clustered with *B. orbignyanus* (UFB: 100, GCF: 80; SCF: 48), and *S. affinis* was grouped with *S. brasiliensis* (UFB: 100, GCF: 100; SCF: 72.9). This agrees with previous report based on *cytb* and *cox1* [14]*,* according to which *B. moorei* is phylogenetically related to the cis-Andean congeners closely related to *Salminus*. Several other authors have also previously proposed the close relationships among *Brycon* and *Salminus* [14, 20, 48-51]. Despite this close phylogenetic relationship, *Salminus* can be unequivocally recognized using alternatively morphological [18] and molecular characters [14, 52, this study]."

P. 15, L. 338-345: The authors should not use the word “migration” which has a very different biological sense, and use instead “expansion” as Abe et al. (2014) did.

We have reordered this section including recommendations from Reviewer #2. Please see below.

References (only the ones not cited in the MS)

Lima, F.C.T. 2017. A revision of the cis-andean species of the genus Brycon Müller & Troschel (Characiformes: Characidae). Zootaxa, 4222(1): 1-189
<https://doi.org/10.11646/zootaxa.4222.1.1>

Machado, C.B., Galetti Jr., P.M. & Carnaval, A.C. (2018) Bayesian analyses detect a history of both vicariance and geodispersal
in Neotropical freshwater fishes. Journal of Biogeography, 45, 1313–1325.
<https://doi.org/10.1111/jbi.13207>

Reviewer #2: I Congratulate the authors on the interesting investigation on such important fishes. Overall the manuscript is well-written, but need clarification on several aspects detailed below.

The main one involves the number of sampled species on the paper. Along most of the manuscript 11 species are mentioned, but the figures and the tables show 13, and 19 species are mentioned at least once on the text. This must be thoroughly reviewed.

We assembled 15 mitogenomes from 11 Bryconidae species, but the comparative mitogenomics and evolutionary relationships were analyzed using 19 mitogenomes from 13 species, as we included four previously reported mitogenomes. The title of table 2 was also corrected: “Table 2. Summarized mitogenomic and assemble characteristics of the 13 Bryconidae species.”

On the scientific aspects of the paper, most of my criticism is directed towards the Phylogenetic portions of the manuscript. Not only the presentation of systematic, taxonomic, and biogeographic interpretations of the results obtained seem to be confusing, but also because the phylogenetic analyses and their developments correspond to a secondary portion of the paper that the entire proposal of phylogenetic investigation seem misleading.

Please, see the edited paragraphs below.

On the other hand, I do agree with the authors’ intention perform a comparative/phylogenetic evaluation of the Mitogenomes obtained. Therefore, to reduce any bias presented in the title and objectives of the paper, I would suggest changing the title of tha manuscript to “Comparative evaluation of the mitochondrial genomes of thirteen members of the Bryconidae (Ostariophysi; Characiformes)”, which would represent more accurately the aspects of the paper.

Following the recommendations of Reviewer #1, we have edited the title of the manuscript and indicated explicitly the limitations of our study.

Now:

In the title: “Exploring the mitochondrial genomes and phylogenetic relationships of trans-Andean Bryconidae species (Actinopterygii: Ostariophysi: Characiformes)”

In Discussion: “Since only 19 mitogenomes corresponding to 13 species of Bryconidae were included in our analysis, the phylogenetic relationships found may be influenced by an incomplete sampling. Despite this limitation, the current analysis recovers the phylogenetic relationships previously reported and offers new insights into the origin and diversification of Bryconidae groups.”

In addition, I have found instances where the text has some sort of problems:
Lines
2 - Authors should include taxonomic indexers after Bryconidae, to locate readers from other fields on the branch of the tree of life the paper is aiming.

Now: “Exploring the mitochondrial genomes and phylogenetic relationships of trans-Andean Bryconidae species (Actinopterygii: Ostariophysi: Characiformes)”

21-22 - A phylogeny including 11 among 50 species is far from bridging a gap, especially if a previous study has presented a hypothesis with more than half the diversity of the species of the group which is barely explored in the manuscript

Now in Discussion: “Since only 19 mitogenomes corresponding to 13 species of Bryconidae were included in our analysis, the phylogenetic relationships found may be influenced by an incomplete sampling. Despite this limitation, the current analysis recovers the phylogenetic relationships previously reported and offers new insights into the origin and diversification of Bryconidae groups.”

23 - the number of species comprised in the study is confusing, the authors mention 11 here and elsewhere in the text, but the tables show 13 species instead. this information should be reviewed and if the numbers are correct, clear sentences on the result section explaining which information or analyses apply to 11 or 13 species.

As it was indicated above, we assembled 15 mitogenomes from 11 Bryconidae species, but the comparative mitogenomics and evolutionary relationships were analyzed using 19 mitogenomes from 13 species, as we included four previously reported mitogenomes.

33 - The term “lack of monophyly” seems innappropriate as monophyly is not “present or absent” in a group. Instead, a group is monophyletic or not.

Now: “Since the current members of *Brycon* are split in three separated lineages, the systematic classification of Bryconidae requires further examination.”

189 - table 2 caption mentions 19 species, when 13 are presented.

Done. Now: “Table 2. Summarized mitogenomic and assemble characteristics of the 13 Bryconidae species.”

251-252 - see comments above.

282-283 - this sentence is confusing, as it seems the authors are comparing the mitogenomes of Salminus and Bryconids with other groups among the examined, which is not accurate. I suggest rewording.

Now, we include the word “previously” to emphasize that the studies referenced [36-40] are not part of our study.

“The overall AT skews (0.015 - 0.071; Median: 0.057) indicate a greater AT bias compared to that previously observed in Characidae [36-40]”

321-322 UFB and Bootstrap are used as synonyms, but UFB would fit better the methods presented in the paper.

Done

326-328 this sentence should be merged with the previous one, concluding that Salminus is actually a species of Brycon with several modifications.

For clarity, we have edited this section: “Furthermore, *B. moorei* was found to be clustered with *B. orbignyanus* (UFB: 100, GCF: 80; SCF: 48), and *S. affinis* was grouped with *S. brasiliensis* (UFB: 100, GCF: 100; SCF: 72.9). This agrees with previous report based on *cytb* and *cox1* [14]*,* according to which *B. moorei* is phylogenetically related to the cis-Andean congeners closely related to *Salminus*. Several other authors have also previously proposed the close relationships among *Brycon* and *Salminus* [14, 20, 48-51]. Despite this close phylogenetic relationship, *Salminus* can be unequivocally recognized using alternatively morphological [18] and molecular characters [14, 52, this study]."

329 change “monophyletic grouping of Bryconidae” to “monophyly of Bryconidae”. At the same time, with two outgroups, that is not actually at the reach of the current results.

Done

329-337 this entire paragraphs lacks logical or theoretical cohesion: the “validity of trans-andean Brycon” has absolutely no connection with their close relationship with Chilobrycon or the fact that they occur in the pacific slope. Are the authors suggesting that those species need to be accommodated in Chilobrycon as well? Although I agree with the suggestion, this discussion is taxonomic, being mixed with biogeographical inferences along the rest of the paragraph, and it should be reorganized in distinct portions of the text.

Now, we reordered these ideas:

“This mitochondrial phylogenomic analysis supports the monophyly of Bryconidae and *Salminus*. However, as reported by other authors, current members of *Brycon* are split in separated lineages [14, 49]. Interestingly, members from Pacific slopes drainages are more closely related to the monotypic *Chylobrycon* than the remaining congeners, corroborating the need to revise the taxonomy of trans-Andean *Brycon*. A plausible alternative would be to expand the current concept of *Chylobrycon* to include *B. chagrensis*, *B. henni*, *B. meeki* and *B. oligolepis*, but this action requires a further comprehensive taxonomic and morphological revision.

Based on the similar topology with a previous study [14], one general hypothesis that can be drawn based on this mitochondrial phylogenomic analysis is that the diversification of the ancestor of Bryconidae originated in north-western South America, followed by vicariant events that isolated the Pacific clade, which subsequently invaded Central America. The hypothesis suggesting a potential invasion of Central America by Bryconidae, as previously proposed [14], aligns well with the notion of a stepwise colonization of *Hyphessobrycon* from the Pacific slope of northwestern South America to middle America [54]. This reinforces the need for continued investigation and exploration to refine the historical biogeography and evolutionary dynamics understanding within the Bryconidae family.

The expansion of Bryconidae in South America was proposed by Abe et al. (2014) [14] based on the hypothesis of López-Fernández and Alberts (2011) [52] according to which substantial marine regressions in the Oligocene, akin to earlier periods, revealed extensive interior floodplains, a scenario that is believed to have expedited the rapid expansion of freshwater habitats. The common ancestor of the clade that includes *B. rubricauda*, *Salminus*, and the remaining *Brycon* species suggests that the expansion occurred from northwestern towards the eastern and southern South America. This hypothetical scenario should be examined in future biogeographic studies using mitochondrial and nuclear markers with a wider taxonomic and geographic representation.”

346 - add article “by the Bryconidae”

Done

350-351 “the Bryconidae family” is a redundance as all taxa ending in -idae are in the family rank.

Done

6. PLOS authors have the option to publish the peer review history of their article ([what does this mean?](https://journals.plos.org/plosone/s/editorial-and-peer-review-process#loc-peer-review-history)). If published, this will include your full peer review and any attached files.


If you choose “no”, your identity will remain anonymous but your review may still be made public.


**Do you want your identity to be public for this peer review?** For information about this choice, including consent withdrawal, please see our [Privacy Policy](https://www.plos.org/privacy-policy).

Reviewer #1: No

Reviewer #2: **Yes:** Andre Luiz Netto-Ferreira
